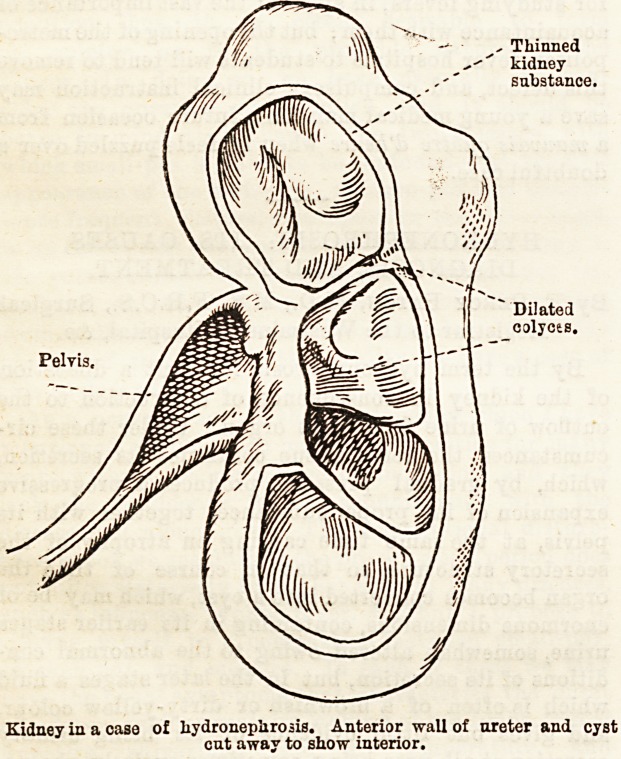# Hydronephrosis: Its Causes, Diagnosis, and Treatment

**Published:** 1896-08-22

**Authors:** E. Percy Paton

**Affiliations:** Surgical Registrar to the Westminster Hospital, &c.


### HYDRONEPHROSIS: ITS CAUSES, DIAGNOSIS, AND TREATMENT.

By E. Percy Paton, M.D., M.S., F.R.C.S., Surgical Registrar to the Westminster Hospital, &c.

By the term hydronephrosis is meant a dilatation of the kidney in consequence of obstruction to the outflow of urine from that organ. Under these circumstances the renal tissue continues its secretion, which, by gradual pressure, produces a progressive expansion of its proper substance, together with its pelvis, at the same time causing an atrophy of the secretory structure, so that in course of time the organ becomes converted into a cyst, which may be of enormous dimensions, containing in its earlier stages urine, somewhat altered owing to the abnormal conditions of its secretion, but in the later stages a fluid which is often of a brownish or dirty-yellow colour, and gives but little evidence of its being urinary secretion at all, urea being sometimes entirely absent, owing to the extensive destructive alteration of the secreting portion of the organ, while traces of blood and albumen can usually be found. A minor degree of this condition is not infrequently found in the post-mortem room, when quite unsuspected during life. In these cases it is due usually to a partial interference with the urinary outflow, which often acts on both kidneys, though usually one is more dilated than the other. Such causes are, tumours of the female genital organs, enlarged prostate, stricture of the urethra, &c. But in most of them the kidney is not enlarged enough to be noticeable in the abdomen as a swelling, and the hydronephrotic condition of that organ is a trouble of only very secondary importance. There are other conditions, however, which, acting on one kidney only, cause at times complete obstruction to the escape of urine, while at other times a certain amount of fluid may be permitted to escape, in which case the kidney soon begins to show itself as a tumour, and to give rise to other troubles.

These causes may be divided into congenital and acquired. The former are those in which some abnormality of the urinary organs exists, such as irregular origin of the ureter from the kidney on one side, kinks and folds of the ureter either through its whole substance or in its mucous membrane or icongenital narrowing of its lumen, &c. Probably the most common acquired cause is impaction of a stone in the ureter, but the same result is sometimes produced by tumours or inflammatory swellings in the pelvis or in the bladder, while kinking of the ureter from partial twisting of the pedicle in a moveable kidney will give rise to the same result. Complete total obstruction of the ureter does not seem to give rise to a very large cyst, such as occurs when the fluid can occasionally escape, at any rate to some small extent.

Kidney in a case of hydronephrosis. Anterior wall of ureter and cyst cut away to show interior.

The symptoms of this trouble are often very slightly marked, the earliest things sometimes noticed being an increase in the size of the abdomen, with the development of a palpable tumour. In some cases, however, very severe pain may accompany the formation of the swelling; this pain is not usually constant, but comes on in paroxysms, which are at times very violent and similar in nature to renal colic, and is, no doubt, in some cases, due to the presence of a stone, though not in all. In these cases the pain is at times accompanied by vomiting, and occasionally the cyst is found to be more tense at the time of greatest pain. A most typical symptom, and one which when present settles the diagnosis, is the occasional disappearance of the tumour, followed shortly after by the passage of a large quantity of urine of rather low specific gravity, with possibly a trace of albumen.

The physical signs in the earlier stages are those of a renal tumour containing fluid. This tumour is usually best felt by the bimanual method; that is to say, by pressing forward the swelling by one hand on the loin, while the other hand examines it through the abdominal wall. At this stage, too, a vertical band of resonance over the tumour indicates that the colon is lying over its anterior aspect. The tumour, on deep inspiration, is found to move little, if at all, on descent of the diaphragm. As, however, the cyst increases in size, the colon is pushed over towards the middle line, and the tumour gradually extends until it may become so large as to occupy the major portion of the abdominal cavity, and, by pushing up the diaphragm, seriously impede the breathing.

The diagnosis of these cases may be easy or difficult. In the early stages, before much enlargement has taken place, a mistake is usually not of much importance. The question at this point will probably be as to, first, whether the swelling is really in the kidney or outside of it, in which latter case it is most usually pus, forming a perinephric abscess, and pushing the kidney forwards, when there will usually be febrile constitutional symptoms with deep œdema and tenderness in the loin, calling for an incision which will then settle the question. A swelling of the kidney itself which seems fluid may be really a soft, rapidly-growing, new growth, when, however, there is usually a considerable quantity of blood in the urine; it may also be a pyonephrosis, in which case there will be pus in the urine, which is usually still acid in reaction, or if the ureter is obstracted it is merely a hydronephrosis which has suppurated. Or it may be hydatids if the kidney, which practically cannot be certainly diagnosed before operation, unless hydatids are found elsewhere, or hooklets or parts of cyst wall are found in the urine.

When the tumour is of large size there is usually a somewhat urgent call for active treatment, and any error in diagnosis may be serious. The most probable mistake, which has been not infrequently made, and by very competent surgeons, is to look upon the case as one of ovarian cyst. This error, however, may be usually avoided by noting the direction of growth of the tumour if this can be got from the history, which, if it be kidney, will have commenced in the loin, and if ovarian will have risen from the pelvis. A vaginal examination will also often help greatly by allowing portions of the cyst if it be ovarian to be felt down in the pelvis, or if the cyst has only extended upwards into the abdominal cavity the uterus will most probably be drawn up with it, or its relations otherwise altered.

Another mistake which is perhaps even easier to make is to confuse the swelling with a largely distended gall bladder. Here, however, the cyst will be more rounded or pyriform in shape, will be more superficial and usually not on the loin at all, and will usually move freely on respiration.

As, however, has been mentioned above, the most typical symptom of a hydronephrotic cyst is sudden diminution in its size with a copious passage of urine, and when thus observed by a competent person the diagnosis is certain.

A hydronephrotic kidney usually calls for active treatment for one of two reasons, namely, its size or the pain it causes. Four different lines of treatment will here be discussed, though practically the last three are really gradual developments in the same direction.

First, manipulation of the tumour has been found in some cases to cause an escape of the fluid, with collapse of the swelling, in a few cases due no doubt to some alteration in some fold of mucous membrane or kink in the ureter, &c. This form of treatment is, however, rarely of any practical use.

Tapping or aspiration is the next resort. This is done by inserting a trocar and cannula, or better, a needle connected with an aspiration apparatus, at a point midway between the last rib and the crest of the ilium, on a line drawn vertically up from a point midway between the anterior and posterior superior spines of the ilium. When the kidney is at all enlarged, at any rate sufficiently so to require tapping, this point will always easily avoid both colon and peritoneum. If, however, one part of the swelling is particularly prominent, it may be tapped at this spot. In most cases tapping, while it gives temporary relief by emptying the cyst, is not permanently successful in curing the trouble, as the cavity speedily refills. Two courses are then open to the surgeon, namely, either to drain the cyst through an incision into it, that is by nephrotomy, or to remove the whole cyst wall and what remains of the kidney by nephrectomy.

Of these plans the better, as a rule, is, at any rate in the first place, to drain the cavity by incision. The operation is easy, consisting in an incision parallel to the last rib, extending from the margin of the erector spinæ muscle outwards for about four inches, and placed about midway between the iliac crest and last rib, the various layers of the lumbar aponeurosis are divided, with some muscle, the cyst exposed, opened, and a tube inserted. When drained in this way, the cavity often rapidly contracts, and the only trouble generally left is that of a sinus in the loin which discharges a urinous fluid, which often becomes quite small in quantity.

Nephrectomy is a much more serious undertaking for various reasons. In the first place it is extremely difficult to obtain any useful idea as to the functional activity of the kidney which will be left behind, and this is more especially the case if the diseased kidney be not first drained to see to what extent it assists in the urinary secretion.

All the various methods of catheterising the ureters are difficult to carry out even in women, and are practically impossible in most cases in men. Even examining the supposed healthy kidney by an abdominal incision does not give any certain information as to its functional activity. If, however, the hydronephrotic kidney is certainly known to discharge no urine into the bladder, a careful qualitative examination of the urine, combined with a knowledge of its quantity and the amount of urea it contains, will give valuable information.

Then, again, if the tumour be large the magnitude of the cyst to be excised adds greatly to the risk, but after it has been drained for a time it will be very much reduced in size, and the subsequent removal, should it become necessary from the trouble given by a sinus discharging a urinous fluid from the loin, will be much less serious. In many cases, indeed, even when there is a discharging sinus left, by means of a well-fitting apparatus this may be made to give so little trouble that the larger operation may become unnecessary. Another advantage of an incision into the tumour before nephrectomy is that it permits of examination as to the cause of the obstruction of the ureter, which, if it turns out to be a removable cause, such as a stone, will obviously do away with the necessity for further operative treatment.

The best line of treatment then is as follows: The tumour may, if desired, be aspirated once, but no sanguine expectations must be entertained that this will cause a permanent cure. Should it refill, or if aspiration be not done, an incision is made into the cyst from the loin, its inside explored with the finger to discover any possible cause, and the cavity subsequently drained. Later, if the sinus left causes trouble, which cannot be comfortably combated by an apparatus to catch the escaping discharge all that remains of the kidney, which will now probably be considerably shrunken, may be excised by an oblique incision on the loin, which may be enlarged by a vertical one at its outer end if sufficient room be not obtained.

## Figures and Tables

**Figure f1:**